# The Entry Lag of Innovative Drugs in Russia, 2010–2019

**DOI:** 10.3390/ijerph18105052

**Published:** 2021-05-11

**Authors:** Alexander Chaplenko, Geliya Gildeeva, Vasiliy Vlassov

**Affiliations:** 1 Department of Organization and Management in the Field of Drugs Circulation, I. M. Sechenov First Moscow State Medical University (Sechenov University), 119435 Moscow, Russia; gildeeva_g_n@staff.sechenov.ru; 2 Department of Health Care Administration and Economics, National Research University Higher School of Economics, 101000 Moscow, Russia; vvlassov@hse.ru

**Keywords:** drug lag, drug regulation, health policy, access to innovative medicines

## Abstract

*Objective:* Evaluation of the lag timelines for the launch of innovative drugs to the Russian market and pharmacoeconomic factors they can depend on. *Methods:* To complete the investigation, we used information about drug products, namely, dates of submission and approval, and pharmacological groups recovered from national registers and official databases. *Results:* Due to impacts of market factors and imperfection of the state regulation, original drugs developed abroad enter the Russian market a few years after their registration in the United States of America, the European Union, and Japan. The average time from the moment of initial approval of a drug in the aforementioned countries and jurisdictions to the moment of registration in Russia is 4 years and 8 months, with a median value of 2.5 years. It has been shown that half of this term is spent on the performance of the procedures of the expertise of the drug registration dossier in the Russian Federation. *Conclusion:* To attain the goal of adequate supplies to the population of the Russian Federation of the most up-to-date, high quality, safe, and efficacious medications, apart from the support of national originators of innovative drugs, we are required to upgrade the existing system of original drug registration. Improvement should be primary focused on the drugs already approved by the leading national regulatory authorities in order to ensure innovative medicine access for Russian patients.

## 1. Introduction 

Implementation of innovative methods of prophylaxis, diagnostics, and disease treatment is one of the key factors of a national healthcare system’s development in any country. Some innovative drugs promise the treatment of illnesses or conditions considered incurable in the past or capable to improve efficacy and safety of currently available therapeutic patterns. 

The effective drug regulatory system should be designed for the market launch of new medications and also should be focused on the increase of accessibility of innovative drugs with clinically proven efficacy. The issue of innovative drug marketing lag has utmost urgency in emerging markets, where the prevailing share of the original drugs are developed by foreign companies. These drugs have already achieved approval in other jurisdictions with bigger markets and better pharmaceutical regulation. The group of such reference jurisdictions includes countries that are the founding regulatory members of the ICH (International Council for Harmonization of Technical Requirements for Pharmaceuticals for Human Use), namely, the USA, the EU, and Japan. As an example of an emerging market, Russia is considered in this article, making a specific emphasis on innovative drugs approved for the sales (registered in accordance with applicable national procedure) in the Russian Federation over the past 10 years.

The drug registration procedure in Russia is, in general, similar to the processes of drug approval in the United States and Japan, as well as marketing authorization in the European Union. In this paper, we will refer to all these processes by the single term “registration”. Applicant submits the drug dossier in the common technical document (CTD) format to the Russian Ministry of Health. Further, the application dossier is assessed in the Scientific Center of Expertise of Ministry of Health. The assessment process includes expertise of the quality of the medicine, as well as the expertise of the “benefit–risk” ratio. The Ministry of Health makes a decision on the registration of the medicine or on the refusal of registration on the basis of the results of the examination. Since 2021, the registration process for pharmaceuticals in the Russian Federation should be carried out in accordance with the regulations of the Eurasian Economic Union, which have not fundamentally changed the registration procedure for original drugs. Therefore, the problems discussed in this study remain relevant today.

## 2. Data Sources and Methods of Analysis

To complete the investigation, we used information about drug products—dates of submission and approval, and pharmacological groups recovered from

− the State Register of Medicines of the Russian Federation (SRM) (http://grls.rosminzdrav.ru/Default.aspx, accessed on 3 March 2021).
− official drug database of the Food and Drug Administration (Drugs@FDA) (https://www.accessdata.fda.gov/scripts/cder/daf/index.cfm, accessed on 15 June 2020).
− European Medicines Agency Register of authorized medicines (https://www.ema.europa.eu/en/medicines/human, accessed on 15 June 2020).
− PMDA (Japan) Register of Review Reports: Drugs (https://www.pmda.go.jp/english/review-services/reviews/approved-information/drugs/0001.html, accessed on 15 June 2020).

We used information about approved drug maximum costs recovered from the State Register of Drugs Maximum Sell Prices of the Russian Federation (http://grls.rosminzdrav.ru/pricelims.aspx, accessed on 15 June 2020).

Data extraction from all the named data sources were finalized on 15 June 2020. Time range of analyzed drugs started on 1 January 2010 and ended on 31 December 2019 for all studied jurisdictions.

Data analysis was conducted in MS Excel 2016 using basic statistical tools and methods. Statistical analysis of values of times between registrations of different types of drugs was conducted using nonparametric Mann–Whitney *U*-test because these values are not distributed normally.

We considered drug as innovative and include in the study if its active moiety was not previously approved for medical use in a particular country.

## 3. Comparison of Circulation Time in the Territory of ICH Countries Prior to Registration in Russia for Different Medication Groups

We evaluated the lag timelines for the launch of innovative drugs to the Russian market using the information recovered from the State Register of Medicines of the Russian Federation (SRM), as well as on data taken from the official websites of pharmaceutical regulatory authorities of the USA, the EU, and Japan.

**Figure 1:**
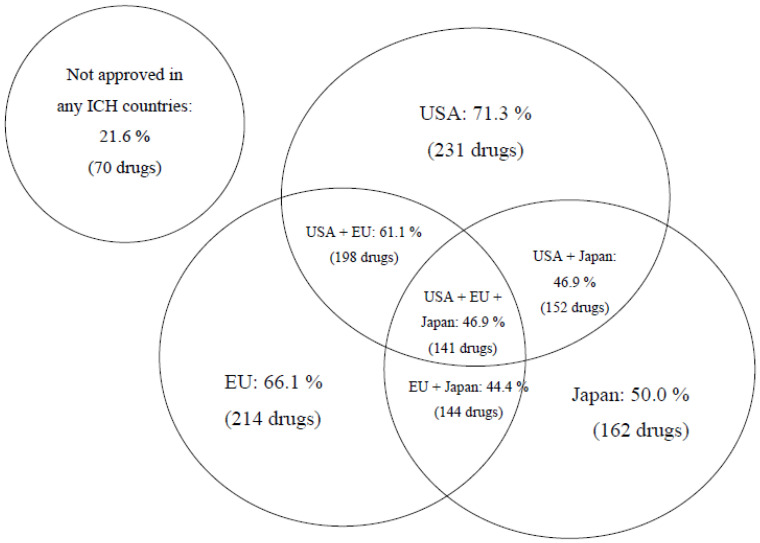
Share of the original drugs registered in Russia for the period of 2010–2019, also approved for use in ICH countries (the USA, the EU, Japan) (as of 31 December 2019). Total number of studied drugs is equal to 324.

We found that 16% of 324 drugs with novel active components registered in Russia in the last decade (2010–2019) have never been approved for use later on in any country worldwide, except for a few members of the Commonwealth of Independent States (CIS) (because of common regulatory principles in Russia and CIS countries), whereas, at the same time, 22% of drugs have not been approved for use in ICH countries (the USA, the EU, and Japan) (see Figure 1 below).

It should be pointed out, however, that the majority (over 78%) of innovative drugs registered in Russia have also been approved for use in at least in one of ICH countries by the time of registration. The delay was one or more years. The share of original drugs registered in Russia later than such medications have been approved in the EU is 97.8%, later than the USA at 93.6% and Japan at 67.6%.

A relatively higher share of drugs registered in the Russian Federation earlier than in Japan is explainable by the mandatory requirement set forth by PMDA national regulatory authority (Pharmaceuticals and Medical Devices Agency). To be registered in Japan, the drug should go through full-scale clinical studies with participation of ethnic Japanese people. This regulatory requirement is based on significant pharmacogenetic differences of the Japanese nation from other ethnical groups, along with the comparative uniformity of the ethnic composition of the Japanese population.

**Figure 2:**
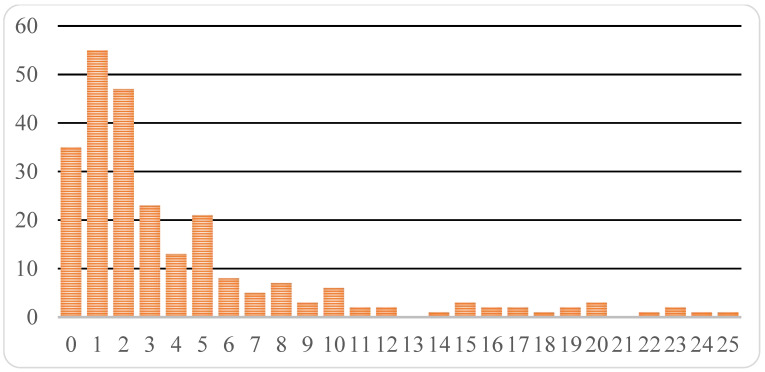
Distribution of innovative drugs registered in the Russian Federation in the period from 2010 to 2019 composed time-wise (in years) and calculated from the moment of the first registration of the drug in the ICH country (ordinates: number of medicines; abscissa: number of complete years after the drug registration in an ICH country to registration in the Russian Federation). Total number of studied drugs is equal to 253.

More detailed analysis of circulation time of innovative drugs before approval in Russia is possible when we combine data from all ICH countries. Two medications from this pool (0.6%): Striverdi^®^ Respimat^®^ (selective β-2 adrenoceptor agonist for bronchial asthma treatment) and Gilenia^®^ (medicine designed for multiple sclerosis therapy) have been registered in the Russian Federation prior to approval in ICH countries, 1.5 years and 1 month earlier for the two drugs, respectively. Average time from the moment of initial registration of a drug in one of ICH countries to the moment of registration in Russia constitutes 1713 days or about 4 years and 8 months. However, the median value is considerably less, just 938 days or 2.5 years. The time distribution is skewed (Figure 2), with some drugs approved in Russia over 10 and even 20 years later.

**Figure 3:**
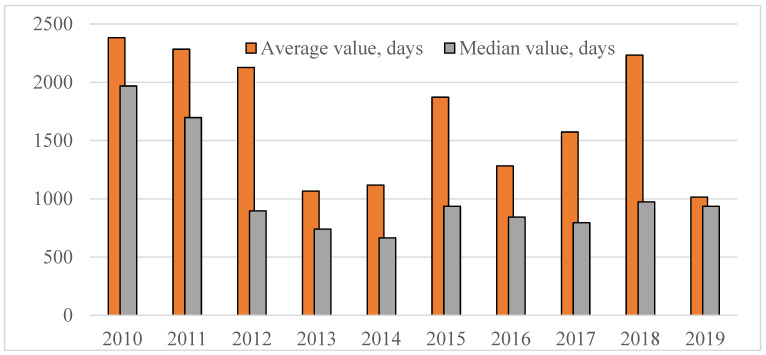
Time (expressed in days) passed from the moment of the first registration of innovative drugs in the territory of an ICH country to the moment of registration in the Russian Federation, depending on the year of the drug’s registration in Russia.

It is important to take into account that analyzed parameter standing for the time passed before drug registration in the Russian Federation is first and foremost defined by two factors: applicant’s motivation to the product launch in the Russian market and duration/complexity of the process of expertise and registration (approval) of innovative drugs in Russia. Let us consider changes in the average time from the moment of the first registration of drugs in the territory of an ICH country to the moment of registration in Russia with year-wise arrangement in the period from 2010 to 2019 (see Figure 3 below).

The figure above shows the trend towards reduction of the median value, which means gradual launch to the Russian pharmaceutical market of drugs being more and more “fresh” from the viewpoint of the international pharmaceutical market. However, starting from 2014, the progress to this matter has experienced considerable slowdown, which could be related to the chill in ties between the Russian Federation and the EU/USA, as well as to contemplation of the draft legislation on the subject of compulsory licensing with regards to holders of rights to original drugs registered in the Russian Federation.

It would also be expedient to analyze the impacts of the drugs’ properties, such as pharmacological activity, cost, and number of potential consumers on the time passed from the moment of registration in ICH countries to the moment of registration in Russia.

To compare registration time of medications with different pharmacological activity, we selected the ATC groups reviewed earlier. Average and median values for the times from registration in ICH countries to registration in Russia that apply to the drugs pertinent to various pharmacological groups are shown in Table 1 below.

**Figure 4:**
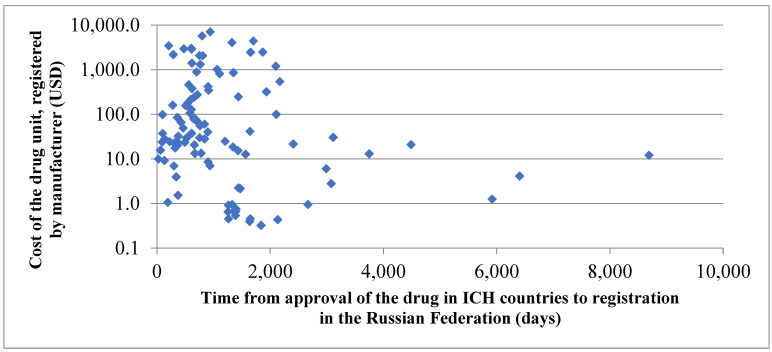
Evaluation of time (expressed in days) passed from the moment of the first registration of innovative drugs in the territory of an ICH country to the moment of registration in the Russian Federation depending on the cost of the drug unit registered in Russia by the manufacturer.

Data in the table above show that the fastest track (in less than 2 years’ time) to the Russian market from ICH countries is covered by medications used to treat diabetes mellitus, as well as by drugs meant for treatment of respiratory system disorders, such as bronchial asthma and chronic obstructive lung disease (COLD), in the first place. The runner-up title is taken by antineoplastic drugs and medicines used for treatment of human immunodeficiency virus (HIV) and hepatitis B/C; these drugs are registered in about 2 years following registration in ICH countries. The longest time is spent by Russian patients in waiting for innovative drugs intended for treatment of musculoskeletal system disorders, dermal diseases, and sensory organs disorders. It is worth pointing out that drugs with the shortest queue for registration are characterized with the same common feature, as they potentially can be indicated to a bigger number of patients. Each of the diseases mentioned above—diabetes mellitus; bronchial asthma; and neoplasms of different localizations, HIV, and viral hepatitis—affect hundreds of thousands of people in the Russian Federation. Moreover, drugs used for treatment of such conditions are generally indicated for the individual’s lifetime, thus resulting in overall cost of the therapy course reaching the level of several tens of thousands (and more often than not hitting hundreds of thousands) of USD. The hypothesis on the relationship between the drug cost and time required for its registration in the Russian Federation (versus registration in ICH countries) could be checked through comparison of registered prices for innovative drugs included in the list of vital and essential drugs (VED). Of 324 original drugs reviewed, the VED list has incorporated 104 (32.1%) items, standing for just under one-third. Figure 4 below shows assessment of the drug cost influence on the pace of its registration in the Russian Federation compared to registration in ICH countries.

Average time from approval in ICH countries to registration in the Russian Federation required for 10% of the most expensive drugs (951 days) was found to be over twofold less than the time required for registration of 10% of the cheapest medicines (2129 days); the difference is statistically significant (*p* ≤ 0.05).

On the basis of the analysis results, we can infer that in the absence of programs for accelerated approval of innovative drugs, it is likely that the national legislation is the principal factor that determines the timeframe for the launch of an original drug to the Russian pharmaceutical market. It stands for economic motivation of holder of rights to the drug, along with a guarantee that the product will generate profits.

Therefore, improvement of the system for legal regulation of innovative drugs’ registration should be focused on support of development and launch to the Russian market of original drugs that are less appealing from the viewpoint of market factors, yet are significant from the clinical perspective, such as new antimicrobial medications, relatively inexpensive remedies, and orphan drugs.

## 4. Main Directions for Development of Control and Permission System

Earlier, we demonstrated the leading role of foreign pharmaceutical companies in the process of development of innovative drugs required to satisfy the needs of domestic medicine. Moreover, we analyzed factors that affect the time gap between registration of original drugs by foreign manufacturers in ICH countries and in Russia.

From this perspective, to succeed in provision of public access to up-to-date high-quality, efficacious, and safe drugs, we must attain the following goals:

- enhancement of motivation of holders of rights to innovative drugs already approved in ICH countries to launch such drugs to the domestic market of pharmaceuticals;
- increase of the number and elevation of quality of Russian pharmaceutical formulations, along with the launch of domestic innovative medications to the local markets of the USA, the EU, and Japan.

Registration of drugs in the Russian Federation is performed in pursuance of declarative procedure [1]. Therefore, it would be of the essence to evaluate what particular barriers of the national control and permission system reduce the incentives of foreign originators already holding approvals issued by FDA/EMA/PMDA in the field of their drugs’ registration in the territory of the Russian Federation.

It would make sense to highlight the barriers of administrative nature (related directly to the procedural and technical parts of drugs’ registration) along with limitations applicable to the contents of submitted documents (associated with the need to additionally confirm quality, efficacy, and safety of drugs).

## 5. Principal Barriers Existing in the Process of Registration of Innovative Drugs by Foreign Originators

At registration stage (in Russia, as well as in ICH countries) the originator is supposed to provide the drug registration dossier, which should contain, apart from administrative documents, relevant information about results of preclinical and clinical development phases, along with the methods used for the drug quality control. Before submission of the drug master file to the regulatory authorities of the Russian Federation, all documents included to the dossier should be translated into the Russian language [2]. Financial outlays and time inputs are insignificant at this stage, and surely do not present a serious obstacle for the drugs access to the market.

Expenses of innovative drug applicants for payment of fees and duties related to marketing authorization issuance and performance of relevant experts’ examinations are minimal in the Russian Federation (less than USD 10,000) [3]. It is especially noticeable when compared to the cost of the services rendered by foreign regulatory authorities (over USD 300,000 in the EU [4] and about USD 3 million in the USA [5]). It should be stressed, however, that there is a mechanism in the USA and EU to grant considerable discounts (up to 98%) for this fee, developed for small-scale research and production companies, as well as for innovative formulations developed for treatment of socially significant and orphan diseases. Supposedly, certain increase of fees and duties level in the Russian Federation combined with implementation of benefits program designed for the original formulations may result in growth of the share of original drugs launched to the Russian pharmaceutical market. Apart from these measures, intended use of duties paid for registration (as is customary abroad) would provide for raising the level and shortening the time required for performance of the pharmaceutical expertise by national regulatory authority.

Another administrative barrier for foreign applicants deals with the need of inspection of foreign manufacturer’s production site by the competent federal body of executive power with the purpose to identify and confirm compliance with the rules of good manufacturing practice [6]. As of today, foreign applicants have only to submit resolution on the subject matter of inspection performance issued by relevant authority (due to limited resources available with inspecting organization). Perhaps, foreign manufacturing sites currently approved by a few regulatory authorities participating in the Pharmaceutical Inspection Cooperation Scheme (PIC/S) should be eligible to a less stringent requirement, keeping up the commitment of the applicant to provide access to the manufacturing site for the Russian inspecting team.

## 6. Harmonization of Requirements for Registration Dossier

The need to modify the structure and composition of registration dossier to submit to the Russian registration authority (the Ministry of Healthcare of the Russian Federation) tends to be minimal, as in pursuance of the last version of Federal Law FZ-61, such a dossier is delivered in the form of Common Technical Document (CTD) adopted in ICH countries. Generally, requirements set forth for the design and scope of conducted preclinical trials and clinical studies have been harmonized. A vital role in the process of harmonization of Russian and international requirements to the registration dossier is performed by integration of the pharmaceutical market of the Russian Federation to the common market of Eurasian Economic Union (EAEU). In the framework of EAEU normative documents’ development, members of the ad hoc committee of Eurasian Economic Commission endeavored to approximate as much as practical the requirements for quality, efficacy, and safety of drugs in the EAEU with relevant requirements existing in the EU [7]. However, absence of the single supranational pharmaceutical regulatory authority gives rise to serious doubts in attainment of true uniformity of the requirements applicable to registration of drugs in the territory of the EAEU.

For instance, entire chapters of Russian guidelines and Articles of Federal Law FZ-61 contravene legislation in effect in the EAEU that permits the acceptance of findings of clinical studies conducted in accordance with the rules of good clinical practice and provisions of the Guideline for Ethnic Factors Evaluation. At the same time, any and all drugs proposed for registration in the Russian Federation, except for orphan drugs (intended for treatment of the diseases with the incidence rate not more than 10 cases per 100,000 subjects), must be clinically studied in the territory of the Russian Federation (including participation in the multicenter international clinical studies). According to part 5 of Article 3 of FZ-61 “*in conformity with the provisions of international treaties and conventions where the Russian Federation is signatory and/or on the basis of reciprocity principle, parties should recognize and accept results of clinical studies of medicinal products for human use conducted beyond the territory of the Russian Federation*”. However, such treaties have never been concluded, the reciprocity principle has not been implemented, and in fact this legal norm is not applied.

Compliance with the requirement to carry out clinical studies of new drugs in the territory of the Russian Federation implies considerable financial expenses and time inputs on the part of the applicant. According to the experts, this issue may present a fundamental problem for a drug launch to the Russian market [8]. On the other hand, results of clinical studies conducted with participation of Russian patients could be more complete in reflection of pharmacogenetic particulars of the population [9]. However, there is a deficiency of Federal Law FZ-61 and other relevant legislative and regulatory instruments from the viewpoint of the requirement to ethnic nationality and citizenship of patients enrolled to the clinical study (formally, a single patient of a Russian hospital included to the multicenter international clinical study will suffice). Along with considerable genetic heterogeneity of the Russian Federation population, it totally rules out the pharmacogenetic sense of the requirement specified above.

A possible argument in support of the requirements set forth for conducting of local clinical studies encompasses compliance with the rules of good clinical practice (GCP), and on the whole, securing plausibility of submitted data on the basis of the clinical study results. It is much easier for the regulatory authority to monitor clinical studies performed locally in the Russian Federation than anywhere abroad. However, at the present moment, there is no regulatory legal mechanism stipulating for revocation of currently valid or for refusal to issue a new marketing authorization for a drug in the situation of detecting unreliable or false data included in the registration dossier (given the case that registration procedure presently in effect in the EAEU implies for performance of pharmaceutical inspections). At any rate, there are no sufficient grounds to assume more rigorous implementation of controls over GCP requirement observance in the Russian Federation, compared to the USA and the EU.

Another positive aspect of imperative conducting of innovative drug clinical studies in the Russian Federation covers the option for some patients to receive innovative drugs on a free-of-charge basis, as well as the opportunity for the healthcare professionals to observe advanced methods of treatment. According to data presented at www.ClinicalTrials.gov accessed on 23 May 2021, Russian clinical facilities are being actively enrolled by major pharmaceutical companies as one of the bases in the scope of multicenter international clinical studies performance, even prior to the first approval for use of drugs in the USA or the EU. Lower cost of conducting clinical studies combined with a higher level of education of the investigators and tremendous interest of physicians and patients make Russia a very attractive site for clinical study performance [10].

## 7. Assessment of Impact of Regulatory Legal Barriers on Drug Registration Time

**Figure 5:**
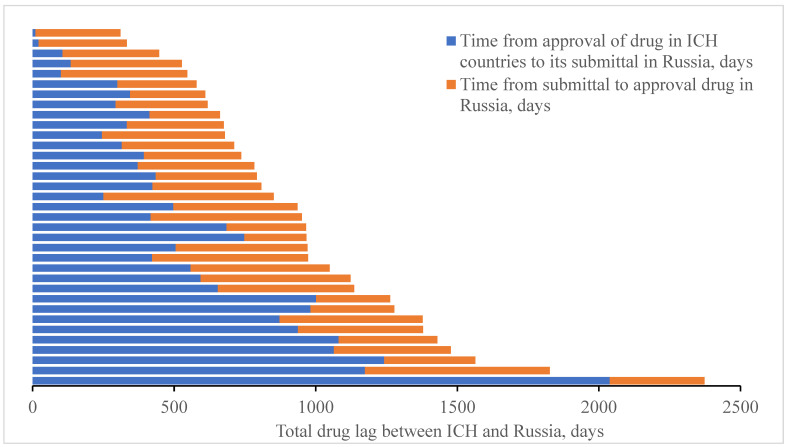
Comparison of time passed from the moment of registration (approval) of original drug in ICH countries to the moment of submittal of application for registration in the Russian Federation and time from submittal of application for registration to actual registration of the drug.

To assess the relationship between delay in drug registration in Russia and lack of applicants’ motivation, as well as to estimate such a relation with existing regulatory legal barriers, we would be expedient to collate the date of registration of the reference number assigned to application for original drug registration (published from 2017 by the Ministry of Healthcare of the Russian Federation at the website of the State Register of Medicines in the “Information Related to State Registration Performance” section) and the date of registration dossier submittal by the applicant in the Russian Federation with dates of registration of the drug concerned in ICH countries and in the Russian Federation. Analysis is provided in Figure 5 below.

It has been shown that with respect to the majority of innovative drugs, duration of registration procedures in Russia exceeds half of the total drug lag time. Therefore, introduction of special programs enabling accelerated registration for essential original drugs including those approved for use in the USA, the EU, and Japan may potentially lead to more than twofold reduction of the time period Russian patients need to spend in waiting for innovative drugs. As an example, one could consider similar programs for accelerated registration of innovative drugs implemented in the USA, the EU, and Japan [11] (refer to Table 2 below).

Today, EAEU legislation has a legal provision setting the option for registration of a generic drug in the territory of the union on the basis of comparison with *“the original drug registered in a country with the level of requirements to the pharmaceutical market regulation not inferior comparing to the level established in the territory of EAEU”*. Obviously, ICH countries should be referred to as such non-inferior states in the first place. In view of this, after successful accomplishment of the expertise of approval for use, a generic drug will be registered in the EAEU, but the original drug used as the basis of comparison for the generic drug registration will necessitate fulfillment of a comprehensive experts’ examination to enable registration in the territory of Eurasian Economic Union [12].

It should be emphasized, however, that implementation of the programs designed for special registration of innovative drugs must be preceded with transparency improvement of the processes of expertise and registration. Lack of publications of expert reports and absence of public discussion of the decisions taken in regard to registration or denial of registration of drugs produce an extremely adverse effect on the attractiveness of the Russian pharmaceutical market for both foreign and domestic originators, manufacturers, and holders of rights to innovative drugs. Integration of clear game rules and compliance with provisions of international legislation applicable in the field of protection of intellectual property rights stand for the key prerequisites for success in increasing affordability and expansion of the range of innovative drugs in Russia. Significant steps in this direction have been taken in the scope of development of legal instruments that regulate the pharmaceutical market of the EAEU. However, it is required that relevant changes to the national legislation be introduced to ensure harmonization with the international rules and standards.

## 8. Conclusions

At present, drug provision for the population of the Russian Federation is secured to a significant extent through involvement of national pharmaceutical companies. In this regard, it should be pointed out, however, that overwhelming majority of the domestic medications stand for reproduced (generic) drugs, being replications of foreign original formulations. To attain the goal of adequate supplies to the population of the Russian Federation of most up-to-date, high quality, safe, and efficacious medications, apart from support of national originators of innovative drugs, we must upgrade the existing system of original drug registration. Improvement should be primary focused on the drugs already approved by the leading national regulatory authorities in order to ensure acceleration of their launch to the civil circulation in the territory of Russia.

Due to impacts of market factors and imperfection of the state regulation, original drugs developed in foreign countries enter the Russian market a few years after their registration in the USA, the EU, and Japan. The average time from the moment of initial approval of a drug in ICH countries to the moment of registration in Russia is 4 years and 8 months, with a median value of 2.5 years. It has been shown that half of this term is spent in performance of the procedures of the drug registration and expertise in the Russian Federation.

Analysis of local experience in the field of original drug registration over the last 10 years allows us to suggest the measures described above, which are aimed at the improvement of the policy currently pursued by the regulatory authority in this area. It should be emphasized that improvement of national legislation in the field of regulation of original drug registration is by no means the only prerequisite to make the quality of drug provision better. It is not enough to register an innovative drug; from that moment onwards, it is required that its affordability to patients is ensured and that mechanisms are developed to prevent emergence of the drug’s shortfall.

## Figures and Tables

**Table 1:** Dependence of the time passed from the moment of innovative drug registration in ICH countries to the moment of registration in Russia on the particular pharmacological group of the drug.

ATC Group	Pharmacological Activity	Average Value (Days)	Median Value(Days)	Number of Drugs
A	Drugs affecting alimentary tract and metabolism	1151.96	807	28
Including A10	Drugs used for treatment of diabetes mellitus	625.23	637	13
B	Drugs affecting blood and blood forming organs	2501.58	1103.5	12
C	Drugs used for treatment of cardiovascular system disorders	1351.67	916.5	12
D	Drugs used for treatment of dermal diseases	3362.67	2857.5	6
G	Drugs used for treatment of the diseases affecting urogenital organs and sex hormones	2590.70	1877	10
H	Systemic hormonal drugs (excluding sex hormones)	3030.40	2019	5
J	Antimicrobial drugs for systemic use	1695.88	969	32
Including J05	Systemic antiviral drugs	1125.25	790.5	20
L	Antineoplastic drugs and immunomodulating agents	1024.06	779	89
Including L01	Antineoplastic drugs	1027.89	779	63
M	Drugs used for treatment of musculoskeletal system disorders	3690.60	3012	5
N	Drugs used for treatment of nervous system disorders	2846.82	2126.5	22
R	Drugs used for treatment of respiratory system disorders	653.50	407	10
S	Drugs used for treatment of sensory organs disorders	3358.33	2854	6
V	Various drugs	3019.90	1972	10
**The average for all groups of drugs**		**1713**	**938**	**253, including drugs from ATC groups not presented in the table**

**Table 2:** Programs for accelerated registration and special procedures of experts’ examination applicable to innovative drugs, in effect in the USA, the EU, and Japan.

Jurisdiction	Program Title	Terms and Conditions for Drug Inclusion	Shortening of Assesment Time	Other Preferences and Benefits
USA	Priority Review	The drug is capable of considerably increasing efficacy and/or safety of the therapy; the drug is intended for treatment of a serious disease	8 months instead of 12	---
	Fast Track	The drug is intended for treatment of a serious disease; preclinical trials or clinical studies have demonstrated the drug’s potential to satisfy unmet medical needs	7 months instead of 12 (if shortening is practicable)	Experts’ follow-up of all stages of development; meetings participated by FDA management
	Breakthrough Therapy	The drug is intended for treatment of a serious disease; preliminary clinical data show significant improvement in comparison with existing methods of treatment		
	Accelerated approval program	Product for a serious or life-threatening disease or condition upon a determination that the product has an effect on a surrogate endpoint that is reasonably likely to predict clinical benefit, or on a clinical endpoint that can be measured earlier than irreversible morbidity or mortality, that is reasonably likely to predict an effect on irreversible morbidity or mortality or other clinical benefit, taking into account the severity, rarity, or prevalence of the condition and the availability or lack of alternative treatments	Not applicable	---
	Emergency use authorization	Unapproved medical products or unapproved uses of approved medical products to be used in an emergency to diagnose, treat, or prevent serious or life-threatening diseases or conditions caused by chemical, biological, radiological, and nuclear threat agents when certain criteria are met, including there being no adequate, approved, and available alternatives	Not applicable	---
EU	Expedited Evaluation	The drug’s significance for healthcare system; innovative character of the drug; availability of rigorous proof of the drug’s efficacy; the drug’s focus on satisfaction of unmet medical needs	150 days instead of 210 (without consideration of the time required for the applicant’s submittal of response to inquiries)	---
	PRIME	Requirements for expedited evaluation + the drug has not been approved in either EU country, and the applicant’s intention to register the drug according to centralized procedure		Appointment of a responsible officer representing Committee on Human Medicinal Products (CHMP) or Committee for Advanced Therapies (CAT) to provide continuous support, arrangement of meetings with experts, joint designing of development plan and regulation strategy, extended scientific consulting
Japan	Priority Review	There is no conventional therapy available or the new drug considerably outscores existing treatments from the viewpoint of the patients’ quality of life, efficacy, or safety; the drug should be intended to treat serious or orphan disease	9 months instead of 12	---
	Sakigake	The drug is oriented to satisfy an unmet medical need; the drug has been developed locally in Japan; preclinical data or the drug’s mechanism of action provide for an assumption of its higher efficacy	6–9 months instead of 12	Priority scientific consulting, appointment of the supervising curator from the Pharmaceuticals and Medical Devices Agency (PMDA), renewal of marketing authorization validity term

## Data Availability

No new data were created or analyzed in this study. Data sharing is not applicable to this article.
